# The impact of fasting and caloric restriction on rheumatoid arthritis in humans: A narrative review

**DOI:** 10.1016/j.clnu.2025.04.025

**Published:** 2025-06

**Authors:** Bérénice Hansen, Marta Sánchez-Castro, Lynn Schintgen, Arefeh Khakdan, Jochen G. Schneider, Paul Wilmes

**Affiliations:** a Luxembourg Centre for Systems Biomedicine, University of Luxembourg, Esch-sur-Alzette, Luxembourg; b Department of Life Sciences and Medicine, University of Luxembourg, Esch-sur-Alzette, Luxembourg; c Department of Microbiome Research and Applied Bioinformatics, Institute of Nutritional Sciences, University of Hohenheim, Stuttgart, Germany; d Department of Internal Medicine II, Saarland University Hospital and Saarland University Faculty of Medicine, Homburg, Germany

**Keywords:** Fasting, Nutrition, Rheumatoid arthritis, Autoimmune diseases, Chronic diseases, Intermittent fasting

## Abstract

Rheumatoid arthritis (RA) is a chronic systemic autoimmune disease affecting approximately 1 % of the global population. It is characterized by swollen and painful joints eventually evolving into bone erosion, cartilage degradation and systemic inflammation, that significantly reduce patients’ quality of life. While modern pharmacological treatments often lead to symptom improvement, they are also accompanied by substantial side effects, which can further impair patient wellbeing.

Dietary interventions, particularly fasting and caloric restriction (CR), have gained increasing attention as adjunctive strategies for RA prevention and treatment. Their anti-inflammatory potential and ability to modulate the gut microbiome render them an attractive option to accompany or modify medical treatment. However, high-quality research on fasting and CR interventions in humans with RA remains limited, and the underlying mechanisms are not yet fully understood.

The present narrative review reflects our current knowledge regarding fasting and CR, emphasising their impact on clinical outcomes, potential underlying mechanism and the sustainability of their effects. Evidence suggests that fasting and CR may lead to short-term improvements in RA disease activity, including reductions in inflammatory markers such as C-reactive protein (CRP) and interleukin-6 (IL-6). However, their long-term efficacy remains uncertain due to the limited duration of most studies. Future research should focus on identifying optimal fasting and CR protocols and their feasibility in long-term disease management, along with investigating patient adherence and potential risks associated with fasting interventions.

## 1 Introduction

### 1.1 Rheumatoid arthritis

Non communicable diseases (NCDs) are the leading cause of mortality in the Western world and their incidence is continuously increasing [1]. Among these, rheumatoid arthritis (RA) stands out as a chronic, systemic autoimmune disease affecting approximately 1 % of the global population and 31.7 million individuals are estimated to be living with RA by 2050 [2,3]. The disease also has a high socioeconomical impact as in addition to indirect and direct medical costs. About 30 % of patients with RA will become work-disabled in the first 2–3 year after their diagnosis [4]. As common in autoimmune conditions, RA shows a pronounced sex disparity: women are three times more likely to develop RA than men, with an increased susceptibility during menopause and the post-partum period [5]. Patients with RA commonly experience severe and chronic pain, stiffness, and other inflammatory comorbidities, which significantly diminish their quality of life [3,6].

The etiopathogenesis of RA is multifactorial and is not yet fully elucidated. Genetic, immunological, environmental, and lifestyle factors contribute to its initiation, progression, and severity. Central to RA pathogenesis is the production of autoantibodies, such as rheumatoid factor (RF) and anti-citrullinated protein antibodies, which trigger the autoimmune recognition of citrullinated proteins in the joints [7]. This process is accompanied by an upregulation of proinflammatory chemokines and cytokines, triggering and perpetuating local inflammation, synovitis and cartilage damage [8].

Immune cell recruitment further exacerbates inflammation. CD4+ T cells, B cells, natural killer (NK) cells, dendritic cells (DCs) and mast cells infiltrate the synovium, releasing various proinflammatory cytokines. Of these, interleukin-6 (IL-6) and tumour necrosis factor- α (TNF-α) play pivotal roles in disease progression and joint destruction [7].A hallmark of the disease is the pro-inflammatory loop, further promoting inflammation and immune system activation in the joints and thereby inducing bone erosion and cartilage degradation [9].

**Fig. 1:**
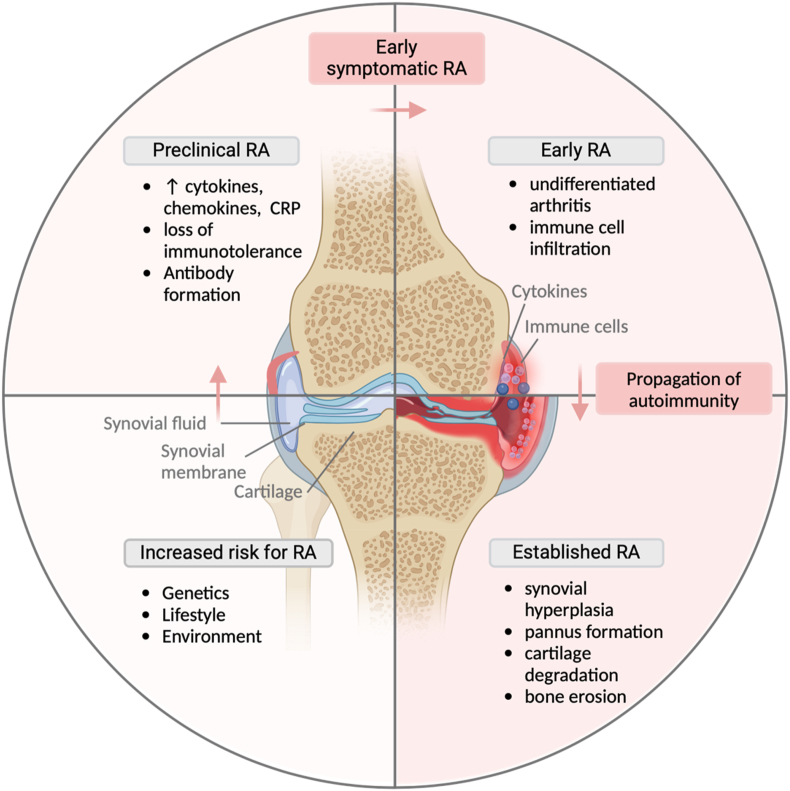
Different stages of rheumatoid arthritis. This figure illustrates the different stages of RA. This schematic outlines the progression of RA from genetic and environmental susceptibility to immune activation and established disease. The arrows indicate progression across phases, from preclinical autoimmunity to clinical onset and chronic joint inflammation. Key processes include the production of autoantibodies, synovial inflammation, and tissue destruction. Adaptive and innate immune cell involvement is also shown, including T cells, B cells, and macrophages. Created in BioRender. Hansen, B. (2024).

Several environmental and lifestyle factors contribute to RA development. Among the most prominent is smoking, which promotes citrullination at local mucosal sites, thereby increasing RA risk [10]. Another significant factor is diet, likely via its influence on the gut microbiome [11]. Dysbiosis, an imbalance in the gut microbiome disrupting the health-promoting harmony of eubiosis, the state of microbial ecosystem balance, is increasingly recognized as a contributor to immune dysfunction in RA [12]. An increase in Firmicutes and Proteobacteria, including *Aggregatibacter actinomycetemcomitans*, *Prophyromonoas gingivalis* and *Akkermansia muciniphila* as well as a decrease in Bacteroides, have been associated with increased RA susceptibility through mechanisms like metabolite secretion, facilitation of citrullination, biomimicry and heightened gut permeability [11,13] (Fig. 1).

Although advancements in pharmacological treatments have significantly improved the management of RA, a substantial proportion of patients remain non-responsive to therapy [14,15]. Moreover, the side effects of current medications, such as increased risk for infections, gastrointestinal side effects or hypercortisolism effects, can further impair quality of life [16]. This underscores the need for complementary therapeutic strategies aimed at symptom alleviation or even disease prevention. Dietary interventions—particularly fasting and caloric restriction (CR)—have emerged as promising approaches due to their ability to confer anti-inflammatory effects and their potential to modulate systemic metabolism and the gut microbiome [17]. Emerging research highlights the potential of these strategies to modulate inflammation, potentially via restoring microbiome balance.

### 1.2 Fasting and caloric restriction in RA

Fasting and CR have gained considerable attention for their potential benefits, particularly their anti-inflammatory effects.

Fasting refers to voluntary abstinence from caloric intake for specific periods, ranging from short-term (intermittent fasting) to prolonged fasting or fasting-mimicking diets, which are increasing in popularity in recent years due to the rising health and wellness culture in the industrialised countries. Notably, fasting differs from starvation. Fasting is intentional and followed by refeeding periods without additional dietary restrictions [18,19]. CR, in contrast, involves a sustained reduction in caloric intake to approximately 70 % of a normo-caloric diet while maintaining sufficient macro- and micronutrient intake to prevent deficiencies. Unlike fasting, CR does not impose temporal restrictions on food consumption (Table 1).

**Table 1:** Different types of fasting and caloric restriction.

Type of fasting	Duration/re-occurrence	Energy intake
Prolonged fasting/Long-term fasting	>4 days – several weeks	200–350 kcal/day
Short-term fasting	2–4 days	200–350 kcal/day
Intermittent fasting	Alternation of fasting periods (≤48 h) and ad libitum food intake	0 kcal alternating with ad libitum
Alternate day fasting	Total fasting or modified fasting on alternate days	0 kcal alternating with ad libitum
Time restricted eating	Periodic total fasting ≥14 h/day	No food intake during fasting, ad libitum during eating phase
Periodic fasting	Any type of fasting repeated at regular intervals	Depends on fasting method applied
Caloric restriction (CR)	Undefined	∼ 70 % of normocaloric intake (avoiding malnutrition)
Fasting mimicking diet (FMD)	5 days of FMD with 1–6 cycles per year	800–1100 kcal

Emerging evidence suggests that fasting and CR exert their beneficial effects through multiple mechanisms. Fasting induces essential metabolic and immunological pathways, that are critical for maintaining homeostasis and adapting to energy scarcity [20]. During regular energy consumption, ATP is primarily produced through glycolysis and subsequent oxidative phosphorylation. However, during fasting, reduced glucose availability triggers the mobilization of energy from adipose tissue and protein stores [20,21]. Due to a decrease in insulin secretion and an increase in glucagon secretion, glucagon receptors on hepatocytes are activated by binding of glucagon, leading to a conformational change and activation of heterotrimeric G protein [22]. This leads to activation of adenylyl cyclase, converting ATP to cyclic AMP (cAMP), cAMP then activates protein kinase A [23]. This, in turn, promotes glycogenolysis and gluconeogenesis. Once glycogen stores are depleted, glycerol derivates from fatty acid (FA) breakdown is converted to glycerol-3-phosphate and then to dihydroxyacetone, which enters glycolysis [24]. This metabolic switch typically begins 10–16 h after the last caloric intake, depending on glycogen reserves and the composition of the previous meal [25].

**Fig. 2:**
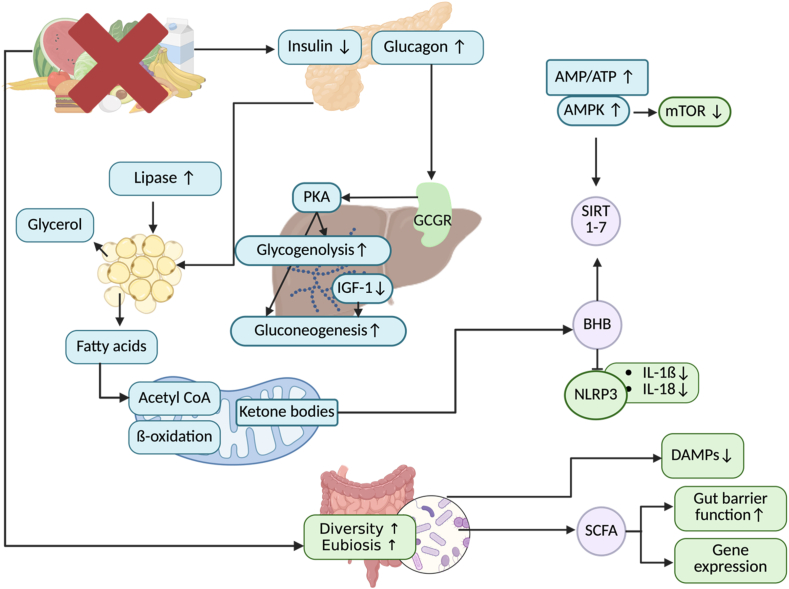
The figure describes the complex underlying mechanisms that have been proposed to mediate the beneficial health effects of fasting. The initial dietary intervention induces a decrease in nutrients triggering the onset of the represented metabolic cascade. The blue boxes illustrate the metabolic switch, the lilac boxes show the induction of several mediators and the green boxes show possible effects induced by the respective key players. AMP, adenosine monophosphate; AMPK, Adenosine monophosphate-activated protein kinase; ATP, adenosine triphosphate; BHB, Beta-hydroxybutyrate; GCGR, glucagon receptor; IGF-1, insulin like growth factor 1; IL, interleukin; DAMPs, damage associated molecular patterns; mTOR, mammalian target of rapamycin; NLRP3, nucleotide-binding domain, leucine-rich–containing family, pyrin domain–containing-3; PKA, protein kinase A; SIRT, sirtuin. Created in BioRender. Hansen, B. (2025) [17]. (For interpretation of the references to color in this figure legend, the reader is referred to the Web version of this article.)

FAs released from the adipose tissue are converted into acyl CoA and subsequently increase the secretion of β-hydroxybutyrate (BHB), a key ketone body [24]. BHB serves not only as an alternative energy source but also as a signalling molecule that regulates the expression of transcription factors such as sirtuins [25]. Sirtuins play vital roles in modulating anti-inflammatory responses, metabolic regulation and protection against oxidative stress, which may explain the several beneficial effects of fasting [26,27] (Fig. 2).

The health benefits associated with fasting are wide-ranging and extend beyond metabolic improvements. They include enhanced mental wellbeing [28,29], neuroprotective effects [30], improved metabolic health [31], symptom reduction in autoimmune diseases due to reduced inflammation [30] as well as anti-cancer properties and support during chemotherapy in humans, due to changes levels of circulating hormones and metabolites amongst others [[28], [29], [30],^32^,^33^]. These effects are likely mediated through fasting's ability to reduce inflammation, restore metabolic balance and modulate immune responses, including those relevant to RA pathophysiology.

Although fasting and CR share overlapping results, such as reduced inflammation and improved metabolic health, their underlying mechanisms differ in several fundamental aspects. BHB, which plays a prominent signaling role during fasting, is not usually elevated in traditional CR, in which calories are moderately restricted without inducing ketosis [34]. This suggests that the anti-inflammatory effects mediated by BHB, including the inhibition of the NLRP3 inflammasome and the modulation of the activity of immune cells, may be exclusive to states of fasting [35]. In contrast, CR may exert its benefits through sustained modulation of nutrient sensing pathways such as AMP-activated protein kinase (AMPK), sirtuins (particularly SIRT1) and inhibition of the mammalian target of rapamycin (mTOR) pathway, contributing to anti-inflammatory and antioxidant responses [36]. These different mechanisms imply that, although both interventions have therapeutic potential in RA, their biological pathways and results may not be interchangeable and require separate clinical evaluation.

## 2 Results

Fasting interventions have emerged as a potential complementary therapy for RA, showing transient but significant benefits on disease activity and inflammatory markers. The studies included in this review (Table 2) consistently demonstrated clear physiological effects in relation to inflammation, metabolic processes, and microbiome dynamics, underscoring its relevance as a possible therapeutic approach. The interventions led to tangible improvements in clinical and inflammatory markers during fasting, reinforcing the reproducibility and reliability of its benefits for RA management. Although four of them utilized overlapping cohorts subjected to fasting interventions, each examined different aspects, such as RA disease activity markers, microbiome changes, and immunoglobulin glycosylation [[37], [38], [39], [40], [41]].

**Table 2:** Summary of studies on RA and fasting regime.

Paper	Intervention	Duration	Participants (n)	Age (mean)	Gender	Outcome
Sköldstam et al., 1979 [47]	Fasting and lactovegetarian diet	7–10 days fasting + 9 weeks lactovegetarian diet	26 (diet: 16 control: 10)	Diet group: 52 (35–66). Control group: 54 (43–65)	19 females (10 diet group, and 9 control group) 7 males (6 diet group and 1 control group)	Fasting led to temporary improvements, no direct microbiome analysis. The lactovegetarian diet had limited effect.
Sundqvist et al., 1982 [42]	Fasting and lactovegetarian diet	10 days fasting + 1-week lactovegetarian diet	10 (diet: 5, control: 5)	Not specified	Not specified	Fasting decreased intestinal permeability and disease activity. No direct microbiome analysis, but suggested a microbiome influence due to improved gut barrier function
Uden et al., 1983 [43]	Fasting + normal food intake (cross-over study)	7 days + 7days	13	42 (24–60)	Females	Fasting reduced joint inflammation, ESR, and improved neutrophil bactericidal capacity. No direct microbiome findings
Kjeldsen-Kragh et al., 1991 [37]	Fasting and vegetarian diet	7–10 days fasting + 3.5 months gluten-free vegetarian diet + 9 months lactovegetarian diet	53 (diet: 27, control: 26)	Diet group: 56 (38–78). Control group: 53 (26–63)	45 females (24 diet group, and 21 control group) 8 males (3 diet group and 5 control group)	Fasting improved disease activity markers (ESR, CRP); vegetarian diet sustained benefits. Controls showed no significant improvements. No direct microbiome analysis.
Peltonen et al., 1994 [38]	Fasting and vegetarian diet	7–10 days fasting + 3.5 months gluten-free vegetarian diet + 9 months lactovegetarian diet	53 (diet: 27, control: 26)	Diet group: 56 (38–78). Control group: 53 (26–63)	45 females (24 diet group, and 21 control group) 8 males (3 diet group and 5 control group)	Significant changes in intestinal flora correlated with RA symptom improvement
Kjeldsen-Kragh et al., 1995 [39]	Fasting and vegetarian diet	7–10 days fasting + 3.5 months gluten-free vegetarian diet + 9 months lactovegetarian diet	53 (diet: 27, control: 26)	Diet group: 56 (38–78). Control group: 53 (26–63)	45 females (24 diet group, and 21 control group) 8 males (3 diet group and 5 control group)	Reduction in *P. mirabilis* antibodies and improvement in disease activity in diet responders
Kjeldsen-Kragh et al., 1995 [48] Based on the abstract. Full article not available.	Fasting and vegetarian diet	7–10 days fasting +3.5 months gluten-free vegetarian diet +9 months lactovegetarian diet	53 (diet: 27, control: 26)	Diet group: 56 (38–78). Control group: 53 (26–63)	45 females (24 diet group, and 21 control group)	Elevated antibody activity against dietary antigens was observed in RA patients, but it did not correlate with clinical outcomes, suggesting food-related immune responses are unlikely to be involved in RA pathogenesis.
Kjeldsen-Kragh et al., 1995 [40]	Fasting and vegetarian diet	7–10 days fasting + 3.5 months gluten-free vegetarian diet + 9 months lactovegetarian diet	53 (diet: 27, control: 26)	Diet group: 56 (38–78). Control group: 53 (26–63)	45 females (24 diet group, and 21 control group)	Decrease in inflammatory markers, leukocyte counts, and complement activity linked to diet. No direct microbiome analysis.
Kjeldsen-Kragh et al., 1996 [41]	Fasting and vegetarian diet	7–10 days fasting + 3.5 months gluten-free vegetarian diet + 9 months lactovegetarian diet	53 (diet: 27, control: 26)	Diet group: 56 (38–78). Control group: 53 (26–63)	45 females (24 diet group, and 21 control group) 8 males (3 diet group and 5 control group)	Decrease in agalactosyl IgG correlated with clinical improvement post-fasting, but not after vegetarian diet period
Fraser et al., 2000 [49]	Fasting or ketogenic diet	7-day fasting vs. ketogenic diet	23 (fasting: 10, ketogenic diet: 13)	Fasting: 49 (31–65); ketogenic diet: 44 (25–69)	Fasting: 9 females, 1 male. Ketogenic diet: 12 females, 1 male	Fasting reduced IL-6 and improved disease activity; both interventions increased DHEAS
Michalsen et al., 2005 [60]	Mediterranean diet vs 8-day intermittent fasting	2 weeks + 3-month follow-up	51 (RA: 16, FM: 35)	49.4 ± 14.3 (MD), 57.7 ± 6.5 (fasting)	MD: 7 females, 0 male. Fasting: 9 females, 0 male	No significant changes in fecal flora or sIgA; clinical improvement in RA observed with fasting (p = 0.09)
Abendroth et al., 2010 [46]	Mediterranean diet and fasting	7 days fasting or MD	50 (fasting: 22, MD: 28)	Fasting: 55.7, MD: 60	Fasting: 21 females, 1 male. MD: 26 females, 2 males	Significant reduction in DAS-28 for both groups, more pain reduction in fasting group. Microbiota alterations were observed with both interventions. Alterations in SCFA in fasting group causing increase of acetate levels.
Hartmann et al., 2023 [61]	Fasting + plant-based diet (PBD) vs anti-inflammatory diet (AID)	7 days (fast) + 11 weeks (PBD) vs 12 weeks (AID)	41 PBD: 24 AID: 17	Not specified	Females	Both diets had comparable impacts on nutrient intake and RA symptoms

Sundqvist et al. [42] reported that a 10-day fasting period significantly reduced disease activity scores (DAS28), including joint inflammation and erythrocyte sedimentation rate (ESR). Similarly, Uden et al. [43] observed substantial clinical improvements in joint status and reductions in ESR during fasting, accompanied by enhanced neutrophil bactericidal capacity, which may contribute to modulating inflammation.

Fraser et al. [44] identified potential immunological mechanisms, involving the immune system, more specifically reporting a 37 % reduction in serum interleukin-6 (IL-6) levels after a 7-day fasting intervention, which correlated with reduced C-reactive protein (CRP) levels and disease activity. Kjeldsen-Kragh et al. [41] further highlighted that fasting significantly reduced agalactosyl IgG levels, a glycoform without terminal galactose from the oligosaccharides on the Fc [45], with these reductions correlating with clinical improvement.

**Fig. 3:**
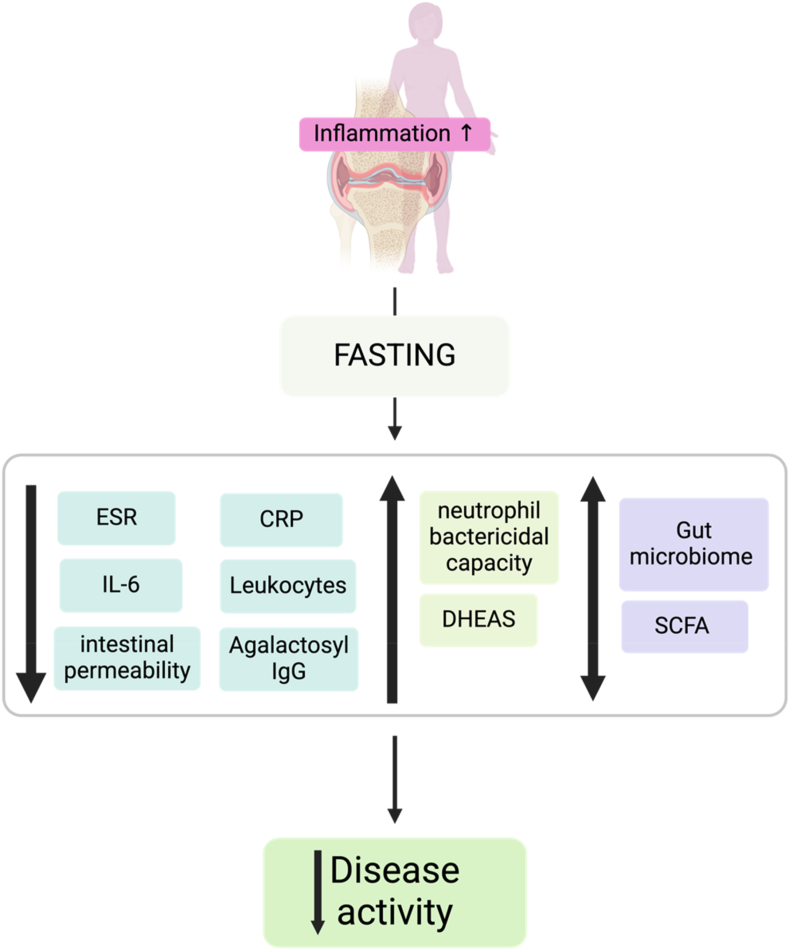
Summary of observed beneficial fasting effects in patients with RA. The figure highlights key outcomes such as reduced levels of inflammatory markers (CRP, IL-6, ESR), modulation of gut microbiota (increase in SCFAs such as acetate), enhanced gut barrier function, and changes in immune signaling (e.g., reduced agalactosyl IgG). Created in BioRender. Hansen, B. (2024).

Microbiome-related changes during fasting were investigated by Peltonen et al. [38] and Abendroth et al. [46], with findings indicating increased acetate levels and shifts in shirt-chain fatty acid (SCFA) profiles, particularly higher levels of acetate in fasting individuals. These microbiome changes were associated with significant clinical improvements, including reductions in the Disease Activity Score (DAS28), joint pain, and ESR, as well as improved visual analog scale (VAS) scores for pain perception. The gut-mediated benefits of fasting could involve several mechanisms, including an enhanced intestinal epithelial barrier integrity, reducing systemic inflammation by lowering the translocation of bacterial endotoxins. Additionally, SCFAs like butyrate and propionate have known anti-inflammatory properties, such as inhibiting nuclear factor kappa B (NF-KB) pathways and reducing pro-inflammatory cytokines like IL-6 and TNF-α. These mechanisms collectively highlight the potential of fasting to modulate gut microbiota and contribute to systemic anti-inflammatory effects (Fig. 3).

In addition to its benefits, fasting is associated with very detrimental few side effects. Gastrointestinal discomfort, including nausea, bloating, and diarrhea, is commonly reported, particularly when preparatory laxatives are used [43,46,47]. Fatigue and weakness are also observed, likely due to caloric deprivation and metabolic adaptations. These effects underscore the need for the close monitoring of patients during fasting interventions.

Kjeldsen-Kragh et al. [48]demonstrated significant reductions in inflammatory markers, including IgM RF, leukocyte count, and complement components C3 and C4, correlating these changes with improved clinical outcomes. Sköldstam et al. [47] confirmed fasting's efficacy in reducing pain, stiffness, and inflammation-related markers, such asα-1-acid glycoprotein, though these benefits were temporary.

## 3 Discussion

Our review synthesizes evidence highlighting fasting's potential to modulate RA symptoms through metabolic, immunological, and microbiome-related mechanisms. The studies consistently demonstrated significant reductions in disease activity markers such as ESR, CRP, and IL-6, accompanied by improvements in clinical symptoms, including reductions in joint swelling, pain, and stiffness [42,43,49]. However, these results should be interpreted with caution due to a variability in study designs and fasting protocols. The studies included in this review varied significantly in terms of fasting protocols, duration, dietary follow-up (e.g. lacto-vegetarian or Mediterranean diets) and patient characteristics such as age, disease severity and gender distribution. The heterogeneity of the included studies (Table 2) complicates direct comparisons between them and may partly explain the differences in the reported results. For example, some studies implemented only a short-term period of fasting, while others combined fasting with months of dietary intervention, which could confuse the effect of fasting with that of plant-based diets. In addition, variations in methodological rigor and sample size further limit the generalizability of the results. Future meta-analyses will require more standardized and homogeneous study designs in order to accurately evaluate efficacy (Table 2).

One of the primary mechanisms of fasting appears to involve the generation of ketone bodies, such as BHB, which not only serve as alternative energy substrates but also function as signaling molecules. BHB potentially modulates inflammatory pathways by inhibiting the NLRP3 inflammasome and upregulating antioxidant responses via nuclear factor erythroid 2-related factor (Nrf2), mostly in the liver and immune cells, amongst others (Fig. 2). Additionally, BHB has been associated with enhanced mitochondrial function, reduced oxidative stress, and altered immune cell activity. These multifaceted yet convergent effects emphasize the critical role of BHB in fasting's anti-inflammatory benefits [44,50]. However, the extent to which BHB accounts for the observed clinical benefits remains uncertain, as the beneficial changes were not observed when following a ketogenic diet [51]. A more complex immunomodulatory mechanism of fasting is suggested, involving systemic adaptations that require more comprehensive exploration.

As previously mentioned, the gut microbiome has gained recognition as a key player in RA pathogenesis. Several opportunistic pathogens identified in patients with RA have been associated with increased secretion of pro-inflammatory cytokines, such as IL-1β, IL-6, and TNF-α, as well as the activation of inflammatory cascades, including the NF-κB pathway [52]. These signaling pathways can be activated by microbial-associated molecular patterns (MAMPs) and danger-associated molecular patterns (DAMPs), such as lipopolysaccharides and reactive oxygen species amongst others. The interplay between these microbial signals and host immune responses further exacerbates systemic inflammation in genetically predisposed individuals [53]. Several studies have proposed fasting-induced alterations in gut microbiota composition, including changes in SCFA production [38,46]. Although the specific microbiota changes vary across studies, the consistent modulation of the gut microbiome highlights fasting's potential as a tool for restoring microbiome balance in RA patients. Dysbiosis-driven disruptions in intestinal homeostasis led to increased permeability, increasing exposure to MAMPs and pathogen-associated molecular patterns (PAMPs). These molecules activate pattern recognition receptors (PRRs), such as toll-like receptors (TLRs), triggering systemic inflammation and contribute towards autoimmune responses. Additionally, DAMPs released during fasting may transiently modulate the immune system and inflammatory processes [54,55]. While promising, the complexity of these interactions underscores the need for advanced studies to dissect causal relationships between microbiome alterations and RA pathogenesis.

In addition to effects directly modulated by the gut microbiome, the observed reductions in agalactosyl IgG and IL-6 during fasting provide insights into fasting's immunomodulatory effects [38,44]. However, these changes were not sustained post-refeeding, underscoring the transient nature of these benefits [41]. This temporary nature highlights a critical limitation of fasting interventions without a maintenance diet. Strategies to sustain these improvements, such as additional dietary and lifestyle adaptations, therefore warrant further exploration.

Despite its benefits, fasting is not without risks. Gastrointestinal discomfort, including nausea, bloating, and diarrhea, as well as fatigue, are frequently reported across studies, particularly during prolonged fasting periods or when preparatory laxatives used [56]. Nutritional deficiencies and rapid weight loss add complexity to fasting's application, especially for vulnerable populations such as those with comorbidities or advanced disease [57]. These issues underscore the importance of careful patient selection, ongoing medical supervision, and individualized intervention strategies to mitigate potential complications [58]. Additionally, fasting can impose psychological stress, manifesting as irritability or mental fatigue, and may create challenges in social contexts where shared meals are integral. Addressing these adherence barriers is essential for the successful integration of fasting into clinical care settings. An individualized approach to fasting interventions may offer enhanced safety and efficacy, particularly for patients with specific metabolic conditions, comorbidities, or medication regimens. Tailoring fasting parameters, such as the timing, duration, and frequency of caloric restriction, should further optimize its benefits while mitigating risks. Furthermore, emerging evidence suggests that personalized fasting protocols could be based on the composition of the baseline microbiome, allowing for targeted modulation of gut dysbiosis and immune responses [[56], [57], [58]].

## 4 Conclusion

Fasting represents a promising complementary therapy for RA, particularly for patients considering the add on of alternatives to pharmacological treatments or experiencing treatment resistance. However, the literature on fasting in RA is scarce and presents mostly short-term studies, with limited exploration of long-term outcomes. Fasting protocols vary widely in terms of duration, dietary composition during refeeding, and overall study design. This lack of standardization complicates comparisons across studies and limits the generalizability of findings. Additionally, many earlier studies relied on less sophisticated analytical methods, leaving significant gaps in our understanding of fasting's molecular and cellular mechanisms. Future research should focus on standardizing fasting protocols and exploring the interplay between fasting, the microbiome, and immunometabolism using advanced techniques such as metagenomics, transcriptomics, and metabolomics to elucidate molecular pathways.

More research is needed to optimize fasting protocols, investigate long-term effects and explore personalized approaches to maximize its therapeutic potential in RA. The currently ongoing ExpoBiome study is studying the effect of prolonged fasting followed by a maintenance diet, consisting of 12 months of time-restricted eating in patients with RA and patients with Parkinson's disease and will elucidate complex underlying mechanisms of this intervention and its beneficial health outcomes [59].

## Author contributions

The authors’ responsibilities were as follows—BH, MSC: conceptualized the research approach, planned and drafted the manuscript outline; BH, MSC: wrote the paper; LS, AK: contributed to literature research; JGS, PW: reviewed and edited the manuscript; and all authors: read and approved the final manuscript.

## Funding statement

This project has received funding from the European Research Council (ERC) under the European Union's Horizon 2020 research and innovation program (grant agreement number 863664). This research was funded in part by the Luxembourg National Research Fund (FNR), grant reference PRIDE/11823097. For the purpose of open access, and in fulfilment of the obligations arising from the grant agreement, the author has applied a Creative Commons Attribution 4.0 International (CC BY 4.0) license to any Author Accepted Manuscript version arising from this submission.

## Conflict of interest

None.
